# Stopping outbreaks with real-time genomic epidemiology

**DOI:** 10.1186/s13073-014-0104-4

**Published:** 2014-11-20

**Authors:** Patrick Tang, Jennifer L Gardy

**Affiliations:** British Columbia Centre for Disease Control, 655 West 12th Avenue, Vancouver, BC V5Z 4R4 Canada; Department of Pathology and Laboratory Medicine, University of British Columbia, Vancouver, BC V6T 2B5 Canada; School of Population and Public Health, University of British Columbia, Vancouver, BC V6T 1Z3 Canada

## Abstract

One of the most successful public health applications of next-generation sequencing is whole-genome sequencing of pathogens to not only detect and characterize outbreaks, but also to inform outbreak management. Using genomics, infection control teams can now track, with extraordinarily high resolution, the transmission events within outbreaks, opening up possibilities for targeted interventions. These successes are positioning the emerging field of genomic epidemiology to replace traditional molecular epidemiology, and increasing our ability to limit the spread of multidrug-resistant organisms.

## Genomic epidemiology for healthcare-associated infections

Healthcare-associated infections (HAIs) are a significant cause of morbidity and mortality in hospitalized patients and represent a major economic burden for healthcare systems. In the United Kingdom, it has been estimated that as many as 300,000 HAIs occur annually at a cost of over £1 billion per year, and that, at any given time, one in every fifteen hospital patients has an HAI [1]. Evidence suggests that approximately 20% of HAIs are preventable [2] and, indeed, HAI point prevalence - the percentage of hospitalized patients with an HAI at any point in time - is decreasing in the United Kingdom, down to 6.4% in 2011 from a high of 9.2% in 1980. However, factors including breakdowns in infection prevention and control practices, unrecognized transmission in the community, and importation of new strains of antimicrobial-resistant pathogens from endemic regions of the world mean that hospitals are regularly seeing the introduction and onward transmission of HAIs in their settings. While surveillance and screening, in combination with molecular genotyping, can indicate the presence of a nosocomial outbreak, conventional molecular epidemiology methods lack sufficient resolution to reveal the origins and transmission dynamics of these outbreaks - information integral to implementing appropriate and effective infection control strategies.

Over the past few decades, a series of molecular epidemiology methods, including pulsed field gel electrophoresis and multi-locus sequence typing, have been developed to estimate phylogenetic relationships between bacterial isolates - each one trying to improve upon the speed, accuracy, reproducibility, ease of use or discriminatory power of previous methods. However, the introduction of next-generation genome sequencing technology has trumped most of these iterative improvements by offering the ultimate in discriminatory power at a relatively low cost. It has the additional benefits of being able to predict antimicrobial resistance phenotypes and identify virulence factors. The potential of this new ‘genomic’ epidemiology for the detection, characterization and management of infectious disease outbreaks, as demonstrated by Pallen and colleagues in this issue of *Genome Medicine* [3], is tremendous. Genomic epidemiology has been instrumental for resolving hospital outbreaks, sometimes disproving previous assumptions regarding nosocomial pathogen transmission. For example, in a recent study of *Staphylococcus aureus* transmission in an intensive care unit (ICU), whole-genome sequencing revealed new transmission events that were missed, and disproved transmission events that were falsely predicted by conventional genotyping [4]. Another study using genomics to study vancomycin-resistant enterococci (VRE) revealed that *de novo* acquisition of vancomycin resistance in *Enterococcus faecium* is probably underappreciated in the hospital environment and that VRE screening at admission may not be sufficient to control VRE within hospitals [5].

## Recent examples of genomic epidemiology in real time

Most genomic epidemiology studies to date have retrospectively analyzed outbreaks, and although this has revealed important insights into pathogen transmission dynamics, the challenge has been to apply genomic epidemiology to directly impact an ongoing outbreak. Only a handful of nosocomial outbreak studies have been performed in real time with the goal of reducing the duration and impact of transmission, including important early work in an outbreak of methicillin-resistant *S. aureus* on a neonatal intensive care ward [6] and a carbapenem-resistant *Klebsiella pneumoniae* outbreak that persisted despite early infection control measures [7].

In 2010, Pallen and colleagues were the first to use whole-genome sequencing to identify a person-to-person transmission event in an infectious disease outbreak, sequencing six isolates of multi-drug resistant (MDR) *Acinetobacter baumannii* from a 2008 hospital cluster to trace transmission between a military and a civilian patient [8]. Now, they report the results of a genomic investigation of a protracted MDR *A. baumannii* outbreak involving a novel strain of the bacterium not previously observed in hospitals in the United Kingdom or other strain collections [9].

The outbreak began with the importation of the novel MDR *A. baumannii* via a military patient, with early secondary cases - linked through conventional molecular epidemiology techniques - occurring on the same ward. At week 40 of what ultimately became an 80-week outbreak, the authors replaced traditional molecular epidemiology methods with whole-genome sequencing, noting that with the less than 1-week turnaround time they achieved with genomics, they were able to more rapidly rule in or rule out isolates as belonging to the outbreak. Of the 102 clinical isolates successfully sequenced, a threshold of less than or equal to 8 single nucleotide variants (SNVs) ruled in 74 genomes as belonging to a single large outbreak, including 52 from individual patient isolates and 10 from environmental sampling.

Phylogenetic analysis of the 74 genomes identified 32 distinct genotypes belonging to seven major clusters. Using a Python script that factors in patient genotype, the ward patients are housed in, and the date of their first positive test, the authors refined the 273 possible transmission events suggested by epidemiology alone to the 57 supported by the genomic data. In this fashion, they established the most parsimonious source of infection for all but 10 patients. The genomic epidemiology suggested that early transmissions occurred through ward-based contact but also through long-term environmental contamination of specific wards, which prompted improved ward decontamination procedures. The genomics also implicated a specific operating theatre for burns patients in several transmissions, leading the infection control team to perform a deep clean of the theatre.

Despite the ward and theatre decontaminations, which had initially appeared to halt the outbreak’s spread, another series of cases occurred from week 70 onwards. The genomic investigation linked the first of these to a contaminated bed, prompting the development of a cleaning protocol specific to this type of bed, with subsequent cases traced again to the burns theatre. Following a second deep cleaning of the theatre, no further transmissions were observed and the outbreak was declared over at week 80.

The real-time use of genomics to reveal transmissions and target infection control interventions to the correct place - be it a ward, operating theatre, or bed - is the most notable aspect of this comprehensive and important work, clearly demonstrating the immediate impact that genomics-informed interventions can have on stopping transmission. It is also worth noting the authors’ use of a software script to develop a putative transmission network - automated approaches can make genomic epidemiology more tractable for infection control teams that may not have specific expertise in interpreting genomic data through the lens of traditional epidemiological relationships.

## What the future holds

With this work, Pallen and colleagues make a convincing case for the utility of whole-genome sequencing as an integral part of infection control practice, demonstrating that it can be done in a clinical setting in real time and that it can lead to evidence-based and effective interventions to stop even a large and protracted hospital outbreak. Given continued advances in technology, such as single-molecule sequencing [10] and bioinformatics methods to resolve mixed infections [11], the accurate and rapid response platform provided by next-generation sequencing will become a cornerstone of infection control. We envision a near future in which hospital laboratories are equipped with genome sequencing technology, enabling pathogen genomes to be derived from direct sequencing of clinical samples, with automated analysis methods to predict drug resistance or to identify clusters of related genomes suggestive of an outbreak. These data will inform the hospital’s infection control program, allowing for real-time evidence-based management of outbreaks, and ultimately decreasing the prevalence of HAIs.

## Abbreviations

HAI: Healthcare-associated infection
MDR: Multi-drug resistant
SNV: Single nucleotide variant
VRE: Vancomycin-resistant enterococci
